# Refined Distances Between Paramagnetic Centers of a Multi-Copper Nitrite Reductase Determined by Pulsed EPR (iDEER) Spectroscopy**

**DOI:** 10.1002/anie.201208166

**Published:** 2013-01-07

**Authors:** Jessica H van Wonderen, Dorota N Kostrz, Christopher Dennison, Fraser MacMillan

**Affiliations:** Henry Wellcome Unit of Biological EPR, School of Chemistry, University of East AngliaNorwich (UK); Institute for Cell and Molecular Biosciences, University of NewcastleNewcastle upon Tyne (UK)

**Keywords:** copper nitrite reductase, EPR spectroscopy, metalloenzymes, structure elucidation

Nitrite reductase from Achromobacter xylosoxidans (AxNiR) is a key enzyme in the anaerobic respiratory pathway of denitrification which catalyses the reduction of nitrite (NO_2_^−^) to nitric oxide.^1^ AxNiR belongs to the large family of multi-copper oxidoreductases^2^ and has been recently shown to have structural similarities to two-domain (small) laccase.^3^ It is a homo-trimer containing six copper ions found in two different geometries.^4^, ^5^ Each monomer contains two distinct copper sites: a buried type 1 (T1) copper site, which receives electrons from external donors, and a type 2 (T2) copper center, located at the monomer–monomer interface, which is the catalytic site where nitrite is bound and reduced to NO (Figure 1). The T1 copper is coordinated by two histidine (His) and a cysteine (Cys) residue in an approximate trigonal planar arrangement, with a relatively weak axial interaction from a methionine (Met) residue (stronger than at most T1 sites). The T2 copper is bound in an approximate tetrahedral arrangement by three His residues, two from one monomer with the third from the adjacent chain, and a water molecule (Figure 1 c). The T1 and T2 copper atoms are 1.24 nm apart in each subunit. The inter-subunit distances between the three T1 Cu centers is 4.35 nm whereas the three T2 Cu centres are 2.96 nm apart. The inter-subunit T1 to T2 copper distances are 3.50 and 3.98 nm (Figure 1 b).

**Figure 1:**
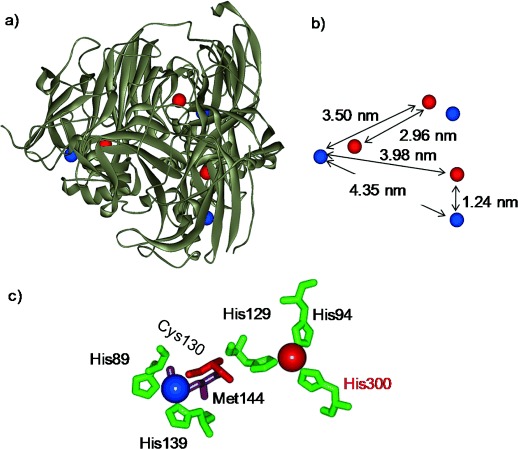
X-ray structure of the AxNiR trimer^5^ with T1 and T2 Cu sites shown as blue and red spheres, respectively. a) Overall architecture of the protein. b) Five different Cu–Cu distances of 1.24, 2.96, 3.50, 3.98, and 4.35 nm present. c) Coordination geometries of the two Cu centers are clearly different.

Pulsed electron-electron double resonance (PELDOR)^6^ distance measurements provide a tool to structurally characterise this enzyme in solution. The protein, as prepared for these experiments, is active at room temperature and in a well-defined redox state, all sites being Cu^II^, which can be advantageous over X-ray structural investigations of single crystals where metal reduction can occur.^7^ Large metalloproteins such as AxNiR can give rise to complex electron paramagnetic resonance (EPR) spectra because of many paramagnetic cofactors and molecular biology, temperature dependence and redox reactivity are often used to simplify and deconvolute spectra. However, it is not always possible to perform certain mutations, redox chemistry is very specific and increasing the temperature can reduce the EPR signal intensity drastically. Another strategy to separate overlapping EPR signals in biological systems uses differences in relaxation rates.^8^ Using pulsed EPR methods, spin–lattice relaxation times (*T*_1_ values) of paramagnetic centers can be determined. If a spectrum possesses two overlapping signals from two centers, the *T*_1_ relaxation times of which are significantly different, then one center can be selectively studied using an inversion-recovery-filtered (IRf) pulsed EPR approach where the signal of the second center is removed by using its intrinsic relaxation time. This methodology has been used previously in combination with hyperfine EPR spectroscopies (REFINE)^8^–^10^ such as ESEEM and HYSCORE.^11^

The presence of multiple paramagnetic centers in AxNiR allows us to further demonstrate the power of PELDOR to study the assembly of proteins. PELDOR, a two-frequency pulse sequence, detects weak dipolar interactions allowing determination of molecular distances (2–8 nm) from these interactions between pairs of paramagnets.^12^ In this work we have developed a new pulse sequence, inversion-recovery filtered (IRf) PELDOR (or iDEER), which combines the inversion-recovery filter (IRf) technique with PELDOR to eliminate either the T1 Cu–Cu or the T2 Cu–Cu distances within the multi-copper AxNiR (see Section S1 in the Supporting Information). This experiment demonstrates that IRf-PELDOR spectroscopy is a powerful tool to study the assembly of proteins that contain multiple paramagnetic centers and adds to the arsenal of distance determination EPR techniques available to structural biologists.

Figure S2-1 depicts the field-swept electron-spin echo (FSE) spectrum of AxNiR at 9.6 GHz. The spectrum is typical of Cu^II^ ions and is due to the presence of T1 and T2 Cu^II^ sites. The four-pulse PELDOR spectra for AxNiR are shown in Figure 2 a. The time trace, in the left panel of Figure 2 a (solid line), is typical of a PELDOR spectrum after background subtraction implemented in DeerAnalysis2008.^13^ This curve was fitted using a distance-domain Tikhonov regularization (dashed line). Figure 2 a (middle panel) depicts the frequency domain spectrum (solid line) and simulation (dashed line). Figure 2 a (right panel) shows that distances of (3.07±0.12), (3.40±0.10), and (4.22±0.16) nm are obtained using a *τ*_2_ of 2600 ns. As a comparison, the distances from the crystal structure (depicted as gray bars)^5^ are shown in Figure 2 f (bottom panel). Using the crystal structure, the distance distribution has been predicted using MMM (Version 2009), a multiscale modelling program of macromolecules,^14^ and is also shown in Figure 2 e (right panel).

**Figure 2:**
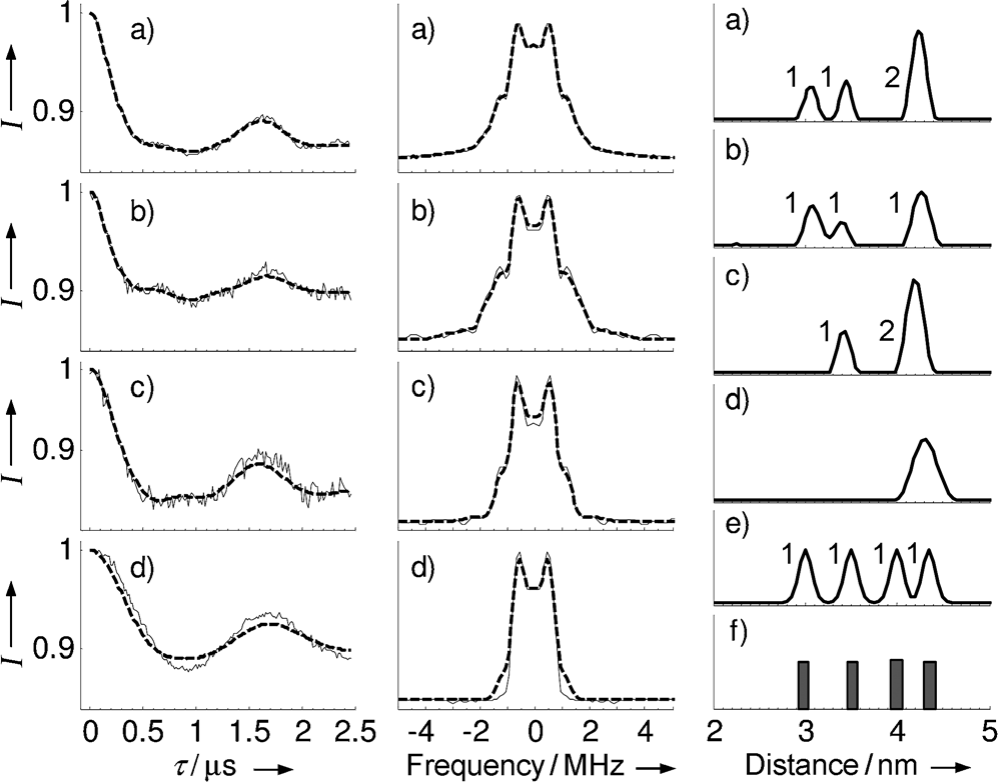
Five-pulse IRf PELDOR performed on AxNiR at about 9.6 GHz and 10 K using the pulse sequence in Figure S1. Filter times of b) 25 (*T*_F_^T1^) and c) 100 (*T*_F_^T2^) μs used and compared to a) no filter. d) Four-pulse ELDOR trace of T2 Cu depleted (T2D) AxNiR. The spectra were recorded at *g*_⊥_ (334 mT) with *ν*_detection_−*ν*_pump_=84 MHz, *τ*_1_=140 ns, and *τ*_2_=2600 ns. The left panels show time traces (solid) and fitted curves (dashed) of spectra after subtraction of the exponential decay. Frequency domain spectra (solid) with simulations (dashed) are shown in the middle panel. Distance distributions are given in the right panel with distances of a) 3.07, 3.40, and 4.22 nm, b) 3.07, 3.37, and 4.24 nm, c) 3.40 and 4.17 nm, and d) 4.28 nm. The Cu–Cu distances derived from the crystal structure are shown as gray bars in (f) and the predicted distances using MMM^14^ are shown in (e). Numbers in the right panels indicate the number of distances assigned to each peak given as a ratio. Analysis was performed using DeerAnalysis2008.^13^

The Cu–Cu distances of 3.07 and 3.40 nm are very similar to those in the crystal structure and from MMM (2.96 and 3.50 nm). The two longer distances in the crystal structure (3.98 and 4.35 nm) are not resolved in the PELDOR data (Figure 2 a, right panel) and appear as a single peak at 4.22 nm with twice the intensity (depicted in Figure 2 a, right panel) labelled by a 2. A longer *τ*_2_ of 4000 ns does not resolve the two longer distances (see Figure S2-1 of the Supporting Information for details). This suggests possible subtle structural differences in solution compared to the single crystal.

The REFINE^8^ method was used to separate the EPR spectra of T1 and T2 Cu^II^ sites and determine their individual filter times, *T*_F_^T1^ and *T*_F_^T2^. By optimizing the filter times to observe only one site, values of 25 and 100 μs, respectively, were determined for the T1 and T2 Cu(II) centers (see Section S2 in the Supporting Information for details).

In addition HYSCORE^11^ experiments at X-band and 10 K (see Figure 3) using the two different filter times were performed to further determine the selectivity for the individual species. The two-dimensional spectra are clearly resolved into regions associated with ^14^N and ^1^H resonances. For I=1/2 nuclei, such as the ^1^H nucleus, the HYSCORE cross-peaks form ridges that have some curvature. Such ridges assigned to the methylene protons of the axial Met ligand at the T1 Cu site are clearly present in the standard HYSCORE spectrum (Figure 3 a, circled).

**Figure 3:**
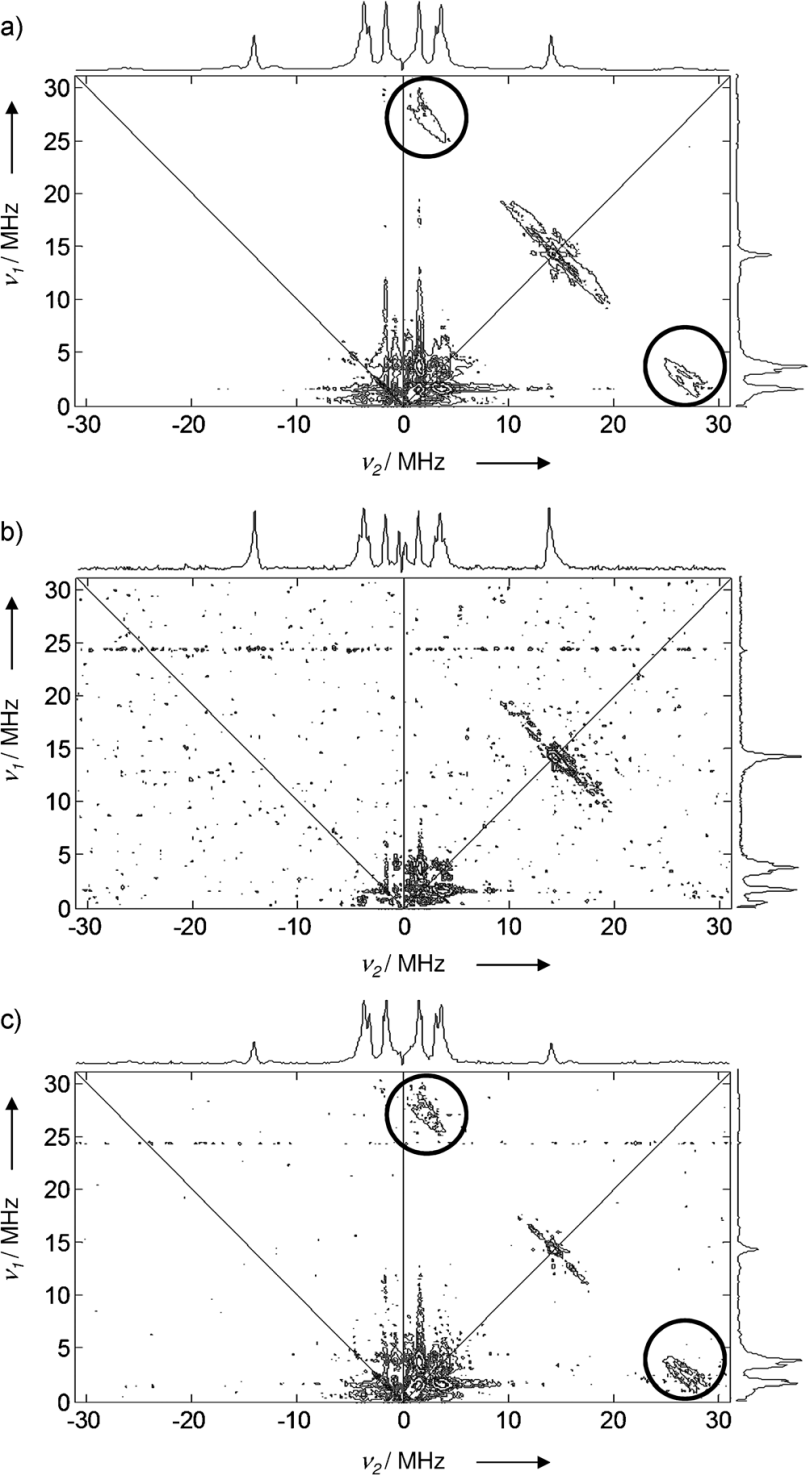
Contour plots of a) 2D-HYSCORE and b,c) REFINE (HYSCORE) spectra of AxNiR. b) Spectrum using *T*_F_^T1^=25 μs; only contributions of T2 Cu are observed. c) Spectrum using *T*_F_^T2^=100 μs; only contributions of T1 Cu are observed. ^1^H peaks, circled, are assigned to hyperfine couplings of β-methylene protons of the methionine residue ligated to the T1 Cu site. In (b) these peaks are not present as the T1 Cu signal is suppressed. Instrumental settings: magnetic field, 333 mT, temperature, 10 K, and *τ*, 132 ns.

In REFINE-HYSCORE spectra acquired using a filter time of 25 μs (*T*_F_^T1^), where only contributions from T2 Cu^II^ ions are observed (Figure 3 b), these ridges are clearly suppressed, whereas using a filter time of 100 μs (*T*_F_^T2^), where only contributions from T1 Cu^II^ sites are expected, these ridges are observed (Figure 3 c). This is a further clear evidence of the selectivity of the applied filter times.

The five-pulse IRf PELDOR sequence is shown in Figure S1 (bottom). The IRf PELDOR spectra are shown in Figure 2 and were recorded at filter recovery times of 25 (b) and 100 μs (c). The IRf PELDOR spectra were also recorded close to *g*_⊥_ at 334 mT using the same *τ*_1_ and *τ*_2_ values as for the regular four-pulse ELDOR (a). The five-pulse ELDOR time traces after a 2nd-order polynomial background correction, performed using DeerAnalysis2008,^13^ are shown in Figure 2 (left panel) for a *T*_F_ of 25 μs (b) and of 100 μs (c, solid lines). The oscillations seen are typical of PELDOR spectra and they are clearly dependent on the filter time. It is clear from the distance distribution (right panel) that, at a *T*_F_ of 25 μs, the T1 Cu signal is suppressed eliminating the T1 Cu–Cu distance at 4.22 nm, resulting in a two-fold decrease in the intensity of this peak (Figure 2 b, right panel). The numbers indicate the intensity ratio of the peaks. Figure 2 d shows the data obtained for a T2 Cu depleted (T2D) AxNiR which shows only one distance of 4.28 nm, comparable to the distance that has been removed in the T1 Cu suppressed sample (b).

At a *T*_F_ of 100 μs, the T2 Cu signal is fully suppressed eliminating the T2 Cu–Cu distance peak at 3.07 nm (Figure 2 c, right panel). So, out of the four Cu–Cu distances, T1-T1, T1-T2, T2-T1, and T2-T2, either the T1-T1 or T2-T2 Cu-Cu distance has been suppressed depending on the filter time *T*_F_ used.

The results can be summarized in terms of having developed a technique which is able to remove an individual distance contribution from a distribution of distances in a biomacromolecule containing multiple paramagnetic centers. The new pulse sequence described is based on the combination of inversion-recovery-filtered EPR and four-pulse ELDOR, which effectively removes single distances from a complex distance distribution. This can be achieved by suppressing the EPR signal of one paramagnetic site (Cu in this case) with an inversion pulse followed by a recovery time that is dependent on the relaxation time of the signal that is being suppressed. As the inversion pulse is only applied at the detection frequency, distances between like centers can be suppressed but distances between unlike centers are still observed.

In complex biomolecules, and especially in oligomeric complexes, with many paramagnetic centers and overlapping EPR signals, PELDOR will give complex distance distributions. By eliminating the distances between pairs of like sites, one at a time, it is possible to simplify and deconvolute the distance distributions. This will be particularly useful when the structure of the protein is unknown.

## Experimental Section

The wild-type enzyme was over-expressed, isolated, and purified, and the T2D protein was prepared, as previously described.^15^ Conventional EPR and PELDOR experimental conditions are given in the Results section and in the figure legends. More details related to the sample preparation and EPR spectroscopy are provided in the text of the Supporting Information.^8^, ^10^, ^13^, ^16^ The pulse sequences for inversion-recovery experiments have been described previously.^8^ The new IRf-PELDOR method is described in detail in the Text S1. For the 5-pulse IRf-PELDOR spectra, a 4-pulse ELDOR sequence was combined with the inversion-recovery filter pulse resulting in a 5-pulse sequence. The same pulse lengths were used (see text in the Supporting Information for details) but now including a 32 ns π inversion pulse at the beginning with a fixed value of *T*_F_ (25 or 100 μs) while incrementing the time *T* in steps of 16 ns. *τ*_1_ and *τ*_2_ were set to 140 and 2600 ns, respectively.

## Notes

As a service to our authors and readers, this journal provides supporting information supplied by the authors. Such materials are peer reviewed and may be re-organized for online delivery, but are not copy-edited or typeset. Technical support issues arising from supporting information (other than missing files) should be addressed to the authors.
