# Supplementary material for: Refined Distances Between Paramagnetic Centers of a Multi-Copper Nitrite Reductase Determined by Pulsed EPR (iDEER) Spectroscopy
Source: Angew Chem Int Ed Engl. 2013 Jan 7;52(7):1990–3. doi: 10.1002/anie.201208166 (PMC3596801; doi:10.1002/anie.201208166)
Supplement: Supplementary file 1 [file anie0052-1990-sd1.pdf]

Angewandte Chemie
Eine Zeitschrift der Gesellschaft Deutscher Chemiker

Supporting Information
© Wiley-VCH 2013
69451 Weinheim, Germany

Refined Distances Between Paramagnetic Centers of a Multi-Copper Nitrite Reductase Determined by Pulsed EPR (*i*DEER) Spectroscopy**

*Jessica H. van Wonderen, Dorota N. Kostrz, Christopher Dennison, and Fraser MacMillan**

anie_201208166_sm_miscellaneous_information.pdf

Table of Contents

S1: Experimental Methods

S2: Additional Results and Figures

S1: Experimental Methods

Protein purification and sample preparation. Wild type AxNiR was over-expressed, isolated and purified as previously described [1] and had a A_{280}/A_{594} ratio of ~11 for the protein fully oxidized with a solution of potassium ferricyanide. Protein concentrations were determined using a molar absorption coefficient of 5200 $M^{-1}cm^{-1}$ at 594 nm.[1] T2 Cu depleted (T2D) AxNiR has the same structure as the wild-type protein but with the T2 Cu removed.[2] The coordination geometry of the type 2 site is almost identical to that found in the wild-type protein. T2D AxNiR was prepared as reported previously [3] except that the incubation time with reductants and chelating agents was shortened to two days. These were removed by overnight dialysis at 4 °C against 100 mM tris (hydroxymethyl) amino-methane (Tris) pH 7.0 under nitrogen followed by dialysis against oxygen-containing buffer. The A_{280}/A_{594} ratio of T2D AxNiR form was ~12 for the fully oxidized form. Protein samples for PELDOR experiments (300 µM) were fully oxidized and were in 20 mM Tris pH 7.5 plus 50 % glycerol.

PELDOR Spectroscopy. X-band pulsed EPR spectra were performed on a *Bruker* E680 spectrometer using a *Bruker* MD5-W1 EPR probehead equipped with an Oxford helium (CF 935) cryostat. The microwave pulses were amplified using a 1 kW-TWT (*Applied Systems Engineering, USA*). All EPR experiments were carried out at 10K. The field-swept spectrum was obtained by integrating the Hahn echo signal as a function of the magnetic field after a two-pulse sequence. The inversion recovery field-swept spectra were obtained as described in [4-6] using the three-pulse (π – T_F – $\pi/2$ – π) sequence and integrating the area under the echo. For inversion-recovery time traces at fixed field positions, all traces are normalized to the echo amplitude without an inversion pulse.

For the 4-pulse PELDOR experiments, pulse lengths were 16 ns for $\pi/2$ and 32 ns for π. The pump pulse length was 30 ns and $\Delta\nu$ ($\nu_{obs} - \nu_{pump}$) was 84 MHz. The pulse separations, τ_1, τ_2, τ_3, were 140, 2600 or 4000 and 100 ns, respectively, and the echo signal were integrated using a video amplifier bandwidth of 20 MHz. The pump pulse was stepped out by 16 ns for a total of 162 points in *T*.

For the 5-pulse IRf PELDOR spectra, a 4-pulse ELDOR sequence was combined with the inversion-recovery filter pulse resulting in a 5-pulse sequence. The same pulse lengths were used as above with a 32 ns π inversion pulse at the start with a fixed value of T_F (25 or 100 µs) while incrementing the time *T* in steps of 16 ns. τ_1 and τ_2 were set to 140 and 2600 ns, respectively.

Spectral Analysis. The Hamiltonian of two interacting spins, $S^1 = ½$ and $S^2 = ½$, in the rotating-frame can be written as [6]:

$$\hat{H}_{12} = \Omega_1 S_z^2 + \Omega_2 S_z^2 + \omega_{ee} S_z^1 S_z^2 \quad (1)$$

where Ω_1 and Ω_2 are the resonant frequencies of spin 1 and 2, respectively, and ω_{ee} is the frequency that represents the coupling between the two spins. Non-secular terms of the spin-spin interaction have been omitted in Equation (1). Neglecting the exchange interaction between electrons and using a point-dipole approximation, ω_{ee} can be expressed as:

$$\omega_{ee} = \frac{\mu_0 g_1 g_2 \mu_B^2}{4\pi\hbar} \frac{1}{r_{12}^3} \left(3\cos^2\theta_{12} - 1\right) \quad (2)$$

where μ_B is the Bohr magneton, g_1 and g_2 are the g-values of the coupled electron spins, ħ is the Planck constant divided by 2π, r_{12} is the distance between the two spins, θ_{12} is the angle between the external magnetic field and the inter-spin vector r_{12}, and μ_0 is the vacuum permeability. As shown in equation (2), the observed dipolar frequency is dependent both on r_{12} and the θ_{12} angle. For the case of nitroxide spin labels, the relative orientations of the two spins are often randomized due to the inherent flexibility of the spin label. The microwave pulses used in a PELDOR experiment can, therefore, uniformly excite most of the θ_{12} values at X-band frequencies. For cases of higher frequencies, restricted spin label motions or Cu(II) PELDOR experiments, the orientations are no longer random and the Tikhonov method [7, 8], which assumes that most θ_{12} angles are excited by the pulses, can no longer be applied. In these cases other methods must be used to analyze the PELDOR data [9]. To overcome this in the Cu(II) experiments described in this work, so that spins from all orientations to the magnetic field are sampled, PELDOR was performed at frequencies close to $g_\perp$ (334 mT) [10], where contributions from molecules with a wide range of orientations are superimposed. This minimizes the effects of orientation selection at these magnetic fields and microwave frequencies and allows Tikhonov regularization to be employed. So, assuming a random relative orientation of r_{12} to the external magnetic field B_0 the distribution of dipolar frequencies ω_{ee} can be described as a Pake pattern [11] with two sharp peaks at:

$$\omega_{ee}^{\perp} = \pm \frac{\mu_0 g_1 g_2 \mu_B^2}{4\pi\hbar} \frac{1}{r_{12}^3} \quad (3)$$

The pulse sequence for a four-pulse ELDOR experiment is described in Figure S1 (top). Two microwave frequencies are used that selectively excite two groups of spins. These are shown as ν_{detect} and ν_{pump}. The spins excited by the detection frequency are referred to as A spins and those excited by the pump frequency as B spins. The initial π and π/2 pulses at the detection frequency excite the A spins producing a Hahn echo. During the evolution time of the Hahn echo of the A spins, the B spins are inverted by the pump pulse. Without the pump pulse, the A spins would evolve in an effective external field with a resonance frequency $\Omega_A \pm \omega_{ee}/2$, where the sign depends on the quantum state of spin B. With a pump pulse, the B spin is flipped, which changes the resonance frequency of the A spin by ω_{ee}, and the time at which that occurs determines how the A spins are refocused by the final π pulse. This causes modulation of the echo, as a function of the time after the echo, at which the pump pulse is applied.

Inversion-Recovery filtered (IRf-) PELDOR Spectroscopy. The pulse sequences for inversion-recovery experiments have been described previously. [4] Figure 2 (bottom) compares the five-pulse IRf PELDOR sequence that combines the IRf technique [4, 5, 12] with four-pulse ELDOR with a conventional four-pulse PELDOR sequence (Figure 2 (top)). The spins excited by the detection frequency are referred to as A spins and those excited by the pump frequency as B spins. An initial inversion-pulse at the detection frequency, ν_{detect}, is applied after which the non-Boltzmann polarization of the electron A spins will relax back to their thermal equilibrium magnetization with a characteristic longitudinal relaxation time T_1.

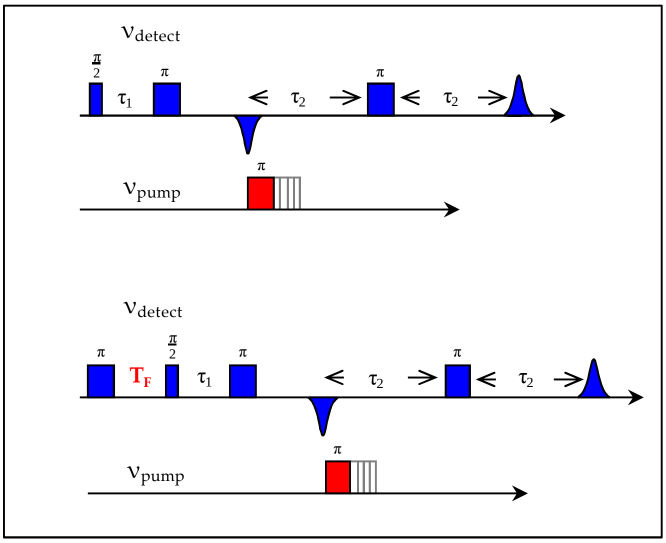

Figure S1. Pulse sequences for PELDOR experiments. (top) Four-pulse ELDOR; (bottom) Five-pulse inversion-recovery filtered (IRf) PELDOR. T_F is the filter time determined by IRf traces and IRf-FSE experiments. See text for experimental conditions.

The B spins are at the pump frequency, ν_{pump}, where the difference in frequency is large enough that they are not affected by an inversion pulse of this length (32 ns). After inversion, the macroscopic magnetization of the A spins decays, traversing a zero-crossing point M_Z. At the time of zero magnetization, T_F, no Hahn echo is observed by the detection sequence, and it is, therefore, suppressed. If there are two or more paramagnetic centers present with different T_1 relaxation times, their spins will each have different filter times, T_F^1, T_F^2 etc. If the four-pulse ELDOR sequence is applied at the T_F time of one of the centers present (A spin), it will be suppressed and will not give an echo. The corresponding B spins, at the pump frequency, will still be excited by the pump pulse as *all* B spins are excited. Hence, when the B spins are flipped, the resonance frequency of the A spins is affected by ω_{ee} to give modulations but only the A spins with an echo will be detected. Therefore, if the A spins of a particular paramagnetic center are suppressed, the dipole-dipole interaction from the B spins of that center are still detected by the A spins of the other center(s) present. So, *only* distances between *like* paramagnetic centers are suppressed by the IRf PELDOR technique.

Systematically suppressing each of the paramagnetic centers present by changing the T_F value and recording IRf PELDOR can simplify the original PELDOR spectrum and help assign the distances obtained. This approach is experimentally tested using a structurally well-characterized system (AxNiR) for which the metal-metal distances are known from X-ray crystallography. Ultimately, this approach will be particularly useful for the analysis of systems for which structural data cannot be obtained from conventional approaches.

S2: Additional Results and Figures

PELDOR of AxNiR. Figure S2-1 (a) depicts the field-swept electron-spin echo (FSE) spectrum of AxNiR at 9.6 GHz. The spectrum is typical of Cu (II) and is due to two types of Cu (II) present, T1 Cu (II) and T2 Cu (II). A simulation performed using Easyspin [13] is also shown in Figure S2-1 (a) (dashed line) using g-values g_{xx} = 2.052, g_{yy} = 2.054 and g_{zz} = 2.196, and a hyperfine constant $A_{||}$ = 230 MHz for the T1 Cu(II) site and T2 Cu(II) parameters of g_{xx} = 2.021, g_{yy} = 2.123 and g_{zz} = 2.341, and a hyperfine constant $A_{||}$ = 400 MHz, which are entirely consistent with previously reported values.[14, 15] The

previously published cw-EPR spectrum of AxNiR also exhibits overlapping T1 and T2 Cu(II) signals, the two lowest field features derived solely from the T2 copper.[14]

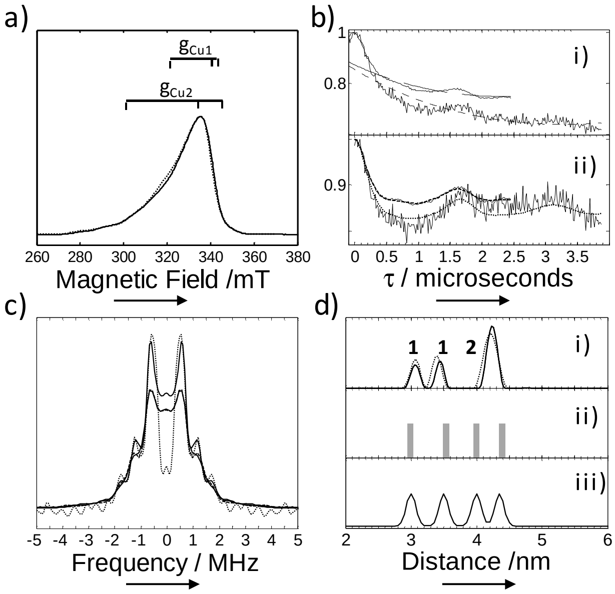

Figure S2-1. A PELDOR experiment performed on AxNiR at ~9.6 GHz and 10 K. a) The FSE spectrum (solid line). A two-pulse echo sequence ($\pi/2$-τ-π) with a 32 ns π-pulse and τ = 140 ns was used. A simulation (dashed line) using Easyspin [13] was performed using the parameters given in the text. b) Four-pulse ELDOR spectrum (i) with τ_2 = 2600 ns or 4000 ns (upper and lower solid lines, respectively) and exponential decays for τ_2 = 2600 ns (dashed line) or 4000 ns (dotted line). The spectra were recorded close to $g_\perp$ at 334 mT as with $\nu_{detection}$ = 9.702 GHz and ν_{pump} = 9.618 GHz where τ_1 = 140 ns. (ii) The time traces (solid lines) and fits for τ_2 = 2600 ns (dashed line) or 4000 ns (dotted line) of the spectrum in i) after subtraction of the exponential decay. c) Frequency domain spectra for data with τ_2 = 2600 ns (dashed line) or 4000 ns (dotted line) of AxNiR with simulations (solid lines). d) (i) Distance distributions with peaks at 3.07 (1), ~3.40 (1) and ~4.22 (2) nm using τ_2 = 2600 ns (solid line) and τ_2 = 4000 ns (dotted line) compared to (ii) which shows Cu-Cu distances derived from the crystal structure (Figure 1 in manuscript). The numbers in parenthesis indicate the number of Cu-Cu distances that the peak should represent given as a ratio. (iii) The predicted distance distribution obtained using MMM.[16] Analysis was performed using DeerAnalysis2008.[8]

The four-pulse PELDOR spectra for AxNiR are shown in Figures S2 (b)-(d). The time traces in Figure S2-1 (b, i), solid lines, are typical of PELDOR spectra and feature clear oscillations superimposed on a decay function. The decay function is fitted using a 2nd order polynomial background correction (Figure S2-1 (b, i), dashed line: τ_2 = 2600 ns, dotted line: τ_2 = 4000 ns) as implemented in DeerAnalysis2008.[8] The result of a background subtraction is shown in Figure S2-1 (b, ii), solid lines, and this curve was fitted using a distance-domain Tikhonov regularization (dashed or dotted line as in i). Figure S2-1 (c) depicts the frequency domain spectra (solid lines) and simulations (dashed or dotted lines as in i). Figure S2-1 (d, i) shows that distances of 3.07 ± 0.12 nm, 3.40 ± 0.10 nm and 4.22 ± 0.16 nm are obtained using a τ_2 of 2600 ns. As a comparison, the distances from the crystal structure (depicted as grey bars) [17] are shown in Figure S2-1 (d, ii). Using the crystal structure, the distance distribution has been predicted using MMM (Version 2009), a multi-scale modeling program of macromolecules [16], and is also shown in Figure S2-1 (d, iii).

The Cu-Cu distances of 3.07 and 3.40 nm are very similar to those both in the crystal structure and from MMM (2.96 and 3.50 nm). The two longer distances in the crystal structure (3.98 and 4.35 nm) are not resolved in the PELDOR data Figure S2-1 (d, i) and appear as a single peak at 4.22 nm with twice the intensity (depicted in Figure S2-1 (d, i) labeled by a 2).

Inversion-recovery filtered (IRf) EPR of AxNiR. The inversion-recovery trace for AxNiR at 334 mT is shown in Figure S2-2 (a) (solid line). At a certain time after inversion the macroscopic magnetization decay traverses a zero-crossing point of the magnetization M_Z. At this time value, T_F, no Hahn echo is observed from this center by the detection sequence. The T1 and T2 copper sites of AxNiR have different T_1 relaxation times and have different zero-crossing point filter times (T_F values). Therefore, only the EPR spectrum of one site is detected if the field-swept spectrum is recorded with the filter time T_F set to the zero-crossing point of the other center.

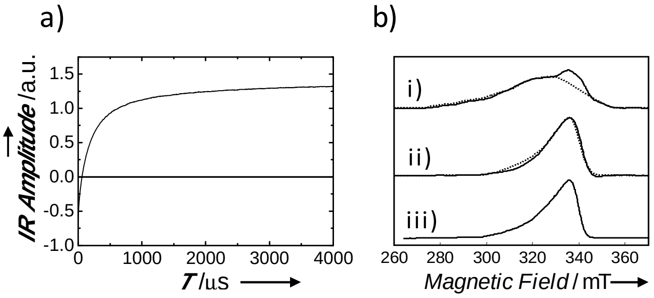

Figure S2-2. Inversion-recovery traces and IRf-FSE spectra of AxNiR at different filter times (T_F). a) Inversion-recovery trace of AxNiR recorded at ~9.6 GHz and 10 K and 3323 G. This is made up of contributions from both the T1 Cu (II) and the T2 Cu (II) species based on their relative contributions to the spectrum at this field value (as determined by spectral simulation). b) Inversion-recovery (IRf) FSE spectra of AxNiR recorded at ~9.6 GHz and 10 K using T_F values of (i) 25 µs and (ii) 100 µs (solid lines). A three-pulse echo sequence (π-T_F-π/2-τ-π) with a 32 ns π-pulse and τ = 140 ns was used. These filter times give the best fits to simulations of (i) T2 and (ii) T1 Cu (II) FSE spectra (dashed lines). The IRf-FSE spectrum with a T_F of 25 µs gave an inverted FSE spectrum so was multiplied by -1 to fit the simulation of T2 Cu (II). In (iii) the FSE spectrum of T2D AxNiR recorded at ~9.6 GHz and 10 K is also shown as a comparison to the T1 Cu (II) in (ii).

To obtain the filter times, T_F^{T1} and T_F^{T2}, for the T1 and T2 Cu (II) sites individually, IRf field-swept spectra were recorded at T_F values between 5 and 500 µs and compared to the individually simulated T1 and T2 Cu (II) spectra that made up the field-swept spectrum of AxNiR shown in Figure S2-1 (a). By optimizing the filter times to observe only one site, values of 25 and 100 µs respectively were determined for the T1 and T2 Cu (II) centers. Figure S2-2 (b) shows the IRf field-swept spectra at T_F^{T1} and T_F^{T2} values of (i) 25 and (ii) 100 µs, respectively (solid lines) with simulations. The field-swept spectrum of a T2 Cu depleted sample of AxNiR (T2D AxNiR) also gives a good fit to the T1 Cu (II) simulation. Suppressing the site with the longer relaxation time (T2 Cu) gives a field-swept signal with intensity 14 % of the maximum intensity. Suppressing the faster relaxing T1 Cu (II) center gives an inverted field-swept signal with an intensity of 10 % of the maximum intensity. Such decreased signal intensity is expected [4] and is compensated for by simply increasing the measuring time in further inversion recovery filtered PELDOR experiments.

[1] K. Sato, C. Dennison, *Chem. Eur. J.* **2006**, *12*, 6647-6659.
[2] E. T. Adman, J. W. Godden, S. Turley, *J. Biol. Inorg. Chem.* **1995**, *270*, 27458-27474.
[3] S. Suzuki, Deligeer, K. Yamaguchi, K. Kataoka, K. Kobayashi, S. Tagawa, T. Kohzuma, S. Shidara, H. Iwasaki, *J. Biol. Inorg. Chem.* **1997**, *2*, 265-274.
[4] T. Maly, F. MacMillan, K. Zwicker, N. Kashani-Poor, U. Brandt, T. F. Prisner, *Biochemistry* **2004**, *43*, 3969-3978.
[5] T. Maly, T. F. Prisner, *J. Magn. Reson.* **2004**, *170*, 88-96.
[6] A. Schweiger, G. Jeschke, *Principles of pulse electron paramagnetic resonance*, Oxford University Press, Oxford, UK ; New York, **2001**.
[7] Y. W. Chiang, P. P. Borbat, J. H. Freed, *J. Magn. Reson.* **2005**, *172*, 279-295.
[8] G. Jeschke, V. Chechik, P. Ionita, A. Godt, H. Zimmermann, J. Banham, C. R. Timmel, D. Hilger, H. Jung, *Appl. Magn. Reson.* **2006**, *30*, 473-498.
[9] J. E. Lovett, A. M. Bowen, C. R. Timmel, M. W. Jones, J. R. Dilworth, D. Caprotti, S. G. Bell, L. L. Wong, J. Harmer, *Phys. Chem. Chem. Phys.* **2009**, *11*, 6840-6848; Z. Yang, D. Kise, S. Saxena, *J. Phys. Chem. B* **2010**, *114*, 6165-6174.
[10] C. W. M. Kay, H. El Mkami, R. Cammack, R. W. Evans, *JACS* **2007**, *129*, 4868-.
[11] G. E. Pake, *J. Chem. Phys.* **1948**, *16*, 327-336.
[12] A. Cernescu, T. Maly, T. F. Prisner, *J. Magn. Reson.* **2008**, *192*, 78-84.
[13] S. Stoll, A. Schweiger, *J. Magn. Reson.* **2006**, *178*, 42-55.
[14] B. D. Howes, Z. H. Abraham, D. J. Lowe, T. Bruser, R. R. Eady, B. E. Smith, *Biochemistry* **1994**, *33*, 3171-3177.
[15] K. Sato, S. J. Firbank, C. Li, M. J. Banfield, C. Dennison, *Chem. Eur. J.* **2008**, *14*, 5820-5828.
[16] Y. Polyhach, E. Bordignon, G. Jeschke, *Phys. Chem. Chem. Phys.* **2011**, *13*, 2356-2366.
[17] M. J. Ellis, F. E. Dodd, G. Sawers, R. R. Eady, S. S. Hasnain, *J. Mol. Biol.* **2003**, *328*, 429-438.
